# Unexpectedly higher levels of anti-orthopoxvirus neutralizing antibodies are observed among gay men than general adult population

**DOI:** 10.1186/s12916-023-02872-0

**Published:** 2023-05-16

**Authors:** Yanmeng Feng, Yifan Zhang, Shengya Liu, Meng Guo, Haojie Huang, Cuiyuan Guo, Wanhai Wang, Wenhong Zhang, Heng Tang, Yanmin Wan

**Affiliations:** 1 Department of Infectious Diseases, Shanghai Key Laboratory of Infectious Diseases and Biosafety Emergency Response, National Medical Center for Infectious Diseases, Huashan Hospital, Shanghai Medical College, Fudan University, Shanghai, 200040 China; 2 Hubei Provincial Center for Disease Control and Prevention, Wuhan, 430065 China; 3 Clinical Laboratory, The First Affiliated Hospital of Zhengzhou University, Key Laboratory of Laboratory Medicine of Henan Province, Zhengzhou, 450052 China; 4 Shenzhen International Travel Health Care Center (Shenzhen Customs District Port Outpatient Clinics), Shenzhen Customs District, Shenzhen, 518033 China; 5 Wuhan Pioneer Social Work Service Center, Wuhan, 430071 China; 6 Shanghai Huashen Institute of Microbes and Infections, 6 Lane 1220 Huashan Rd., Shanghai, 200052 NO China; 7 Department of Radiology, Shanghai Public Health Clinical Center, Shanghai, 201508 China

**Keywords:** MSM, Monkeypox virus, Orthopoxvirus, Preexisting antibodies, Vaccinia

## Abstract

**Background:**

The confirmed cases in the current outbreak of Monkeypox are predominantly identified in the networks of men who have sex with men (MSM). The preexisting antibodies may profoundly impact the transmission of monkeypox virus (MPXV), however the current-day prevalence of antibodies against MPXV among gay men is not well characterized.

**Methods:**

A cohort of gay men (*n* = 326) and a cohort of the general adult population (*n* = 295) were enrolled in this study. Binding antibodies responses against MPXV/vaccinia and neutralizing antibody responses against vaccinia virus (Tiantan strain) were measured. The antibody responses of these two cohorts were then compared, as well as the responses of individuals born before and in/after 1981 (when the smallpox vaccination ceased in China). Finally, the correlation between the anti-MPXV antibody responses and the anti-vaccinia antibody responses, and the associations between preexisting anti-orthopoxvirus antibody responses and the diagnosed sexually transmitted infections (STIs) in the MSM cohort were analyzed separately.

**Results:**

Our data showed that binding antibodies against MPXV H3, A29, A35, E8, B6, M1 proteins and vaccinia whole-virus lysate could be detected in individuals born both before and in/after 1981, of which the prevalence of anti-vaccinia binding antibodies was significantly higher among individuals born before 1981 in the general population cohort. Moreover, we unexpectedly found that the positive rates of binding antibody responses against MPXV H3, A29, A35, E8 and M1 proteins were significantly lower among individuals of the MSM cohort born in/after 1981, but the positive rates of anti-MPXV B6 and anti-vaccinia neutralizing antibody responses were significantly higher among these individuals compared to those of age-matched participants in the general population cohort. Additionally, we demonstrated that the positive and negative rates of anti-MPXV antibody responses were associated with the anti-vaccinia antibody responses among individuals born before 1981 in the general population cohort, but no significant association was observed among individuals born in/after 1981 in both cohorts. The positive rates of both the binding and the neutralizing antibody responses were comparable between individuals with and without diagnosed STIs in the MSM cohort.

**Conclusions:**

Anti-MPXV and anti-vaccinia antibodies could be readily detected in an MSM cohort and a general population cohort. And a higher level of anti-vaccinia neutralizing antibody responses was observed among individuals who did not get vaccinated against smallpox in the MSM cohort compared to age-matched individuals in the general population cohort.

**Supplementary Information:**

The online version contains supplementary material available at 10.1186/s12916-023-02872-0.

## Background

Monkeypox virus (MPXV) is a DNA virus in the Orthopoxvirus genus, which was first discovered in 1959 when cynomolgus macaques were shipped from Singapore to a Denmark research facility [1, 2]. Despite being named after the first described host, monkeys and humans are accidental hosts for MPXV, its natural hosts are more likely to be rodents [3, 4]. MPXV comprises two distinct lineages, the Western Africa strain and the Congo Basin strain. Sporadic human infections by both strains have been reported previously, of which the Congo Basin strain causes more severe disease [5, 6] and accounts for more cases [7].

Compared to previous observations, which showed that the ability of MPXV to transmit among humans was weak [8] and the major clinical manifestations included fever, skin rash and swollen lymph nodes [7], the emerging outbreak is unusual in the following three aspects. First, the current outbreak spreads faster than ever before. Since the first case was reported on May 7, 2022, over 82,000 confirmed cases have been reported (including 65 deaths) by December 14, 2022, spanning 110 nations and regions. Second, it has been shown in the current outbreak that MPXV can present symptoms typical for sexually transmitted infections, such as urethritis, rectal pain and urinary retention [9]. Third, there was a link between MPXV transmission and sexual contact among men who have sex with men [10–13]. Moreover, it is also surprising that the virus causing the current outbreak belongs to the Western African lineage [14], which historically demonstrated low outbreak-causing potential [6, 15].

Epidemiological investigations estimated that the basic reproduction number (R_0_) for MPXV-2022 may be substantially above 1 [16, 17], but the underlying determinants of the outbreak remain elusive [18]. Factors, such as the waning immunity to smallpox [7], genetic evolution of virus [14, 19] and super spreader events [16] have been proposed to contribute to the resurgence and the increased human-to-human transmission of MPXV. Although a modeling study showed that smallpox vaccination conferred protection against all orthopoxviruses [20], it is not the only source of anti-orthopoxvirus immunity. Two previous studies showed that anti-orthopoxvirus antibodies could also be detected in significant percentages of individuals either without previous exposures [21] or with low exposure chances [22]. A more recent study suggested that neutralizing antibodies against MPXV could be detected in individuals without previous vaccination or infection [23]. The preexisting antibodies may profoundly impact the transmission of MPXV, however the current-day prevalence of antibodies against any orthopoxvirus is unknown. To fill the knowledge gap, in this study, we detected and compared the anti-MPXV and anti-vaccinia in an MSM cohort and a general population cohort.

## Methods

### Study populations and sample collection

In this study, we enrolled a cohort of gay men (*n* = 326) who were under routine follow-up at Hubei Provincial Center for Disease Control and Prevention, China and a cohort of the general adults (*n* = 295). Individuals with chronic diseases (tumor, hypertension, coronary heart disease, autoimmune diseases and diabetes) and active infections (Influenza, SARS-CoV-2, HIV, HBV, HCV, tuberculosis, etc.) were excluded. The demographical characteristics of the enrollments were depicted in Table 1.

**Table 1:** Demographical characteristics of enrolled participants

	**Number**	**Age (Median, 25%-75% percentile)**
**General_Man**		
≤ 41 years	46	33.00 (28.00–37.25)
> 41 years	72	53.00 (48.00–60.75)
**General_Woman**		
≤ 41 years	97	31.00 (28.00–36.00)
> 41 years	80	49.00 (45.00–55.50)
**MSM**		
≤ 41 years	321	26.00 (23.00–29.00)
> 41 years	5	48.00 (43.50–52.00)

*MSM* men who have sex with men

Peripheral blood was collected by venipuncture into ethylenediaminetetraacetic acid (EDTA) tubes by medical personnel. After centrifugation, plasma was transferred into cryotubes and stored at − 80 °C until test.

### Vaccinia virus (Tiantan strain)

The vaccinia virus (Tiantan strain) was kindly gifted by Dr. Yiming Shao (State Key Laboratory for Infectious Disease Prevention and Control, National Center for AIDS/STD Control and Prevention, Chinese Center for Disease Control and Prevention, Beijing, China). The stock used for the binding and neutralizing antibody assays was expanded in primary chicken embryo cells, and harvested via rapid freeze–thaw of the infected cells, a typical method for collecting the mature virions [24]. The harvested virus was further purified through 25% sucrose cushion centrifugation and titrated in VERO cells.

### Detection of binding IgG antibodies against MPXV H3, A29, A35, B6, M1, E8 proteins and vaccinia virus A33, A27, H3 proteins

MPXV H3, A29, A35, B6, M1 and E8 proteins were genetically similar with vaccinia virus H3, A27, A33, B5, L1 and D8 proteins, respectively [25]. As vaccinia proteins A27, H3, A33, B5, L1 and D8 are known to contain neutralizing epitopes [26] and immunization with A27, A33, B5, L1, D8 or H3 [27–29] protected against challenge with virulent virus in mice or macaques, we speculated that antibodies against MPXV H3, A29, A35, B6, M1 and E8 might be relevant to protection. In-house enzyme-linked immunosorbent assays (ELISA) were developed to measure binding antibodies against MPXV H3, A29, A35, B6, M1, E8 proteins and vaccinia A33, A27, H3 proteins. Briefly, high-binding 96-well EIA plates (Cat# 9018, Corning, USA) were coated with purified MPXV A35 (Cat# 40886-V08H, Sino Biological, China), A29 (Cat# 40891-V08E, Sino Biological, China), H3 (Cat# 40893-V08H1, Sino Biological, China), M1 (Cat# 40904-V07H, Sino Biological, China), E8 (Cat# 40890-V08B, Sino Biological, China), B6 (Cat# 40902-V08H, Sino Biological, China) and vaccinia virus A27(Cat# 40897-V07E, Sino Biological, China), A33 (Cat# 40896-V07E, Sino Biological, China), H3 (Cat# CSB-EP324943VAA1, Cusabio, China) proteins at a final concentration of 1 µg/ml in carbonate/bi-carbonate coating buffer (30 mM NaHCO_3_,10 mM Na_2_CO_3_, pH 9.6) and incubated overnight at 4 °C. Subsequently, the plates were blocked with 1 × PBS containing 5% skimmed milk for 1 h at 37 °C. Then, 50 μl of 1:50 diluted human plasma was added to each well. After 1-h incubation at 37 °C, the plates were washed with 1 × PBS containing 0.05% Tween20 for 5 times and incubated with 0.5 M NaSCN for 15 min at room temperature. After incubation, the plates were immediately washed for 2 times. Next, 50 μl of an HRP labeled goat anti-human IgG antibody (Cat# ab6759, Abcam, UK) diluted in 1 × PBS containing 5% skimmed milk were added to each well and incubated for 1 h at 37 °C. After another round of wash, 50 μl of TMB substrate (Cat# MG882, MESGEN, China) was added to each well. 25 min later, the color development was stopped by adding 50 μl of 1 M H_2_SO_4_ to each well and the values of optical density at wavelengths of 450 nm and 630 nm were measured using a microplate reader (Cat# 800TS, Biotek, USA).

### Detection of binding IgG antibodies against vaccinia whole-virus lysate

Vaccinia virus (Tiantan strain) suspended in 1 × PBS were boiled for 10 min and the concentration of total protein was quantified using a bicinchoninic acid assay (BCA) kit (Cat# E112-01, Vazyme, China) according to the manufacturers’ instruction. The experimental procedure was the same with the in-house ELISA assay described above, except that the EIA plates were coated with vaccinia whole-virus lysate at a final concentration of 20 µg/ml.

### Plaque Reduction Neutralization Test (PRNT)

The method of PRNT was established according to published literatures [30, 31] with minor modifications. Briefly, test sera were diluted in Dulbecco's modified Eagle's medium (DMEM) supplemented with 1% FBS and mixed with an equal volume of vaccinia virus (Tiantan strain) suspension (20 pfu/100 μL). The final dilution of the serum samples was 1:30. The mixtures were incubated at 37 °C for 1 h. After incubation, the mixtures were transferred onto confluent Vero cell monolayers in 24-well cell culture plates. The plates were incubated at 37 °C for 2 h in a humidified incubator with 5% CO_2_. Next, 200 μl of overlay-medium (DMEM containing 1% FBS and 1.25% methyl cellulose) was added to each well after removing the supernatant. The plates were incubated for 4 days at 37 °C in the incubator. At the end of incubation, the plates were stained with crystal violet and the number of plaques were manually counted. Fetal bovine serum was used as the negative control. All samples were tested in duplicate wells. The viral inhibition ratio was calculated using the following formula: Inhibition activity (%) = (The average plaque number of the negative control wells − the average plaque number of the sample wells) / (The average plaque number of the negative control wells) × 100%.

### Statistical analysis

Statistical analyses were conducted using Graphpad Prism 9 (GraphPad Software, USA). Normality tests were performed before all downstream statistical analyses except the Chi square test. Comparisons between two groups were performed by the method of non-parametric t-test. Differences among multiple groups were compared by the method of one-way ANOVA. The contingency analysis was done using the method of Chi-square test. Correlation analysis was conducted by the method of Pearson correlation. *P* ≤ 0.05 was considered as statistically significant.

## Results

### Comparisons of binding antibody responses against MPXV and vaccinia between an MSM cohort and a general population cohort

To reveal the prevalence of preexisting antibody responses against MPXV in an MSM cohort and a general population cohort, we first measured the binding IgG responses against vaccinia whole-virus lysate and MPXV H3, A29, A35, E8, B6, M1 proteins using in-house ELISA assays. As China ended smallpox vaccination in 1981, we stratified participants in the two cohorts into two age groups respectively, ≤ 41 (born in/after 1981) and > 41 (born before 1981). The negative control assays were performed in wells without antigen coating and the results showed that the background binding was quite low and comparable between the two cohorts (Additional file 1: Fig. S1). The cut-off value was set at 0.13, which was slightly higher than the twofold of the average OD value of all negative control tests (0.0632). No negative control demonstrated an OD value surpassing the cut-off threshold.

**Fig. 1:**
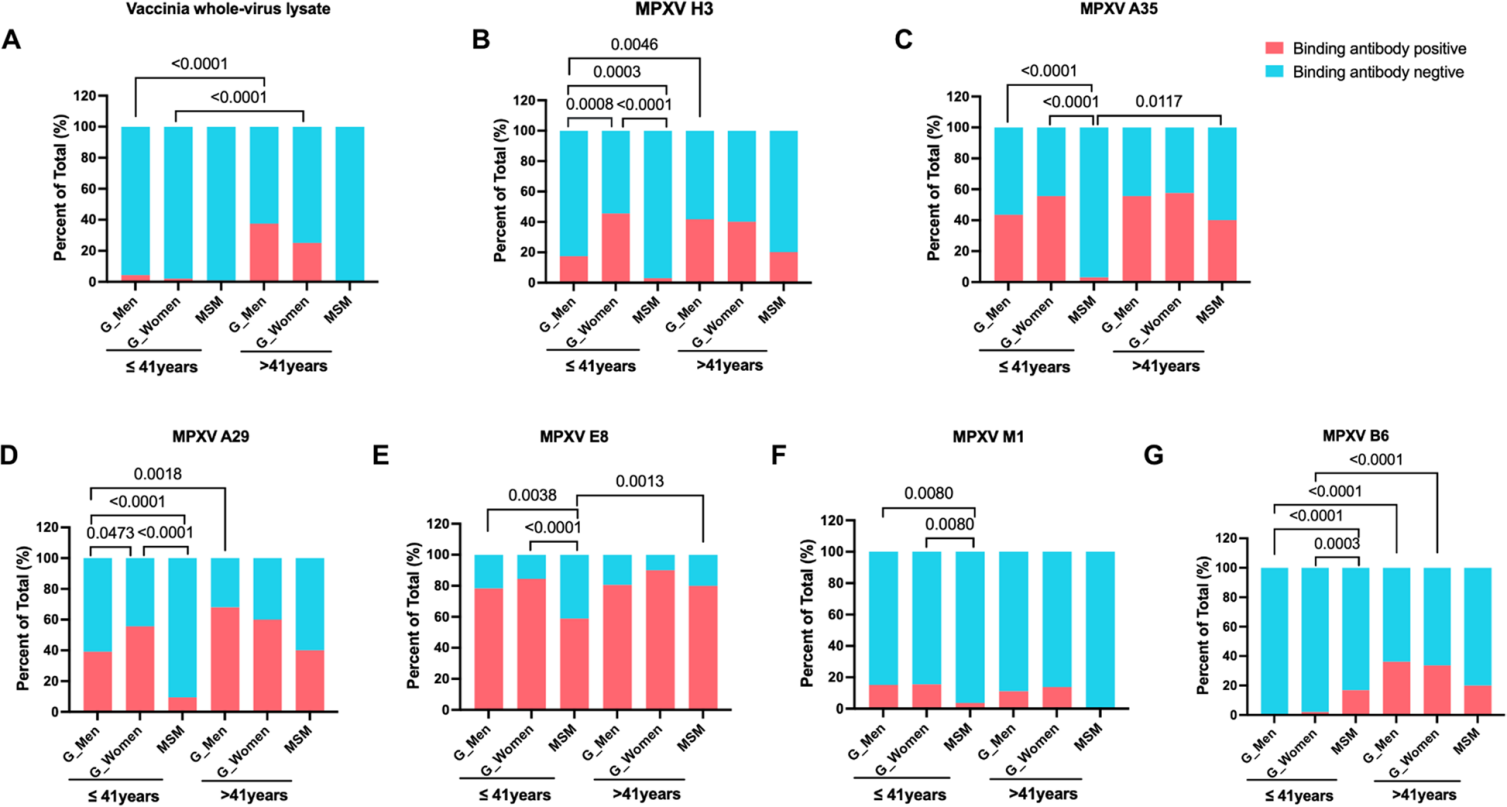
Comparisons of the positive rates of binding IgG responses against vaccinia whole-virus lysate and MPXV H3, A29, A35, E8, B6, M1 proteins. The participants in both cohorts were stratified into two age groups, ≤ 41 years and > 41 years. The participants in the general population group were further separated by sex. The positive rates of binding IgG responses against vaccinia whole-virus lysate (**A**) and MPXV H3, A35, A29, E8, M1, B6 proteins (**B-G**) were compared among groups. Statistical analyses were performed by the method of Chi-square test. G_Men, general men; G_Women, general women

Our data showed that the prevalence of binding IgG responses against vaccinia whole-virus lysate was significantly higher among participants aged > 41 than that among participants aged ≤ 41 in the general population cohort (Fig. 1A), which is in consistence with a previous sero-epidemiological study showing that the prevalence of anti-vaccinia neutralizing antibodies was higher among individuals born before 1980 in China [32]. Most participants (321 out of 326) in the MSM cohort were younger than 41 (Table 1), and age-matched comparisons of the prevalence of anti-vaccinia binding IgG responses showed no significant difference between the MSM cohort and men in the general population cohort (Fig. 1A).

Preexisting binding antibodies against MPXV H3, A29, A35, E8, B6 and M1 proteins were also detectable in both cohorts. The positive rates of binding antibodies against MPXV H3, A29 and B6 were significantly higher among men aged > 41 than those among men aged ≤ 41 in the general population cohort (Fig. 1B, 1D, 1G). The positive rates of antibody responses against MPXV A35 and E8 were significantly higher among participants aged > 41 than those among participants aged ≤ 41 in the MSM cohort (Fig. 1C, E). Age-matched comparisons revealed that the positive rates of antibody responses against MPXV H3, A35, A29, E8, and M1 proteins were significantly higher among participants aged ≤ 41 in the general population than those among participants aged ≤ 41 in the MSM cohort (Fig. 1B-F). Of note, individuals aged ≤ 41 in the MSM cohort showed significantly a higher prevalence of anti-MPXV B6 binding antibody responses than individuals of the same age group in the general population cohort (Fig. 1G). We also compared the OD values of binding antibody assays between the two cohorts. The findings were generally in consistence with the results of prevalence comparisons described above (Additional file 1: Fig. S2).

To verify the results of anti-vaccinia binding antibody responses measured using whole-virus lysate, we detected binding IgG responses against purified H3, A27 and A33 proteins of vaccinia virus. The data corroborated the findings of ELISA assays using whole-virus lysate as the coating antigen (Additional file 1: Fig. S3).

### Significantly higher levels of neutralizing antibody responses against vaccinia virus (Tiantan strain) was observed in the MSM cohort than in the general population cohort

**Fig. 2:**
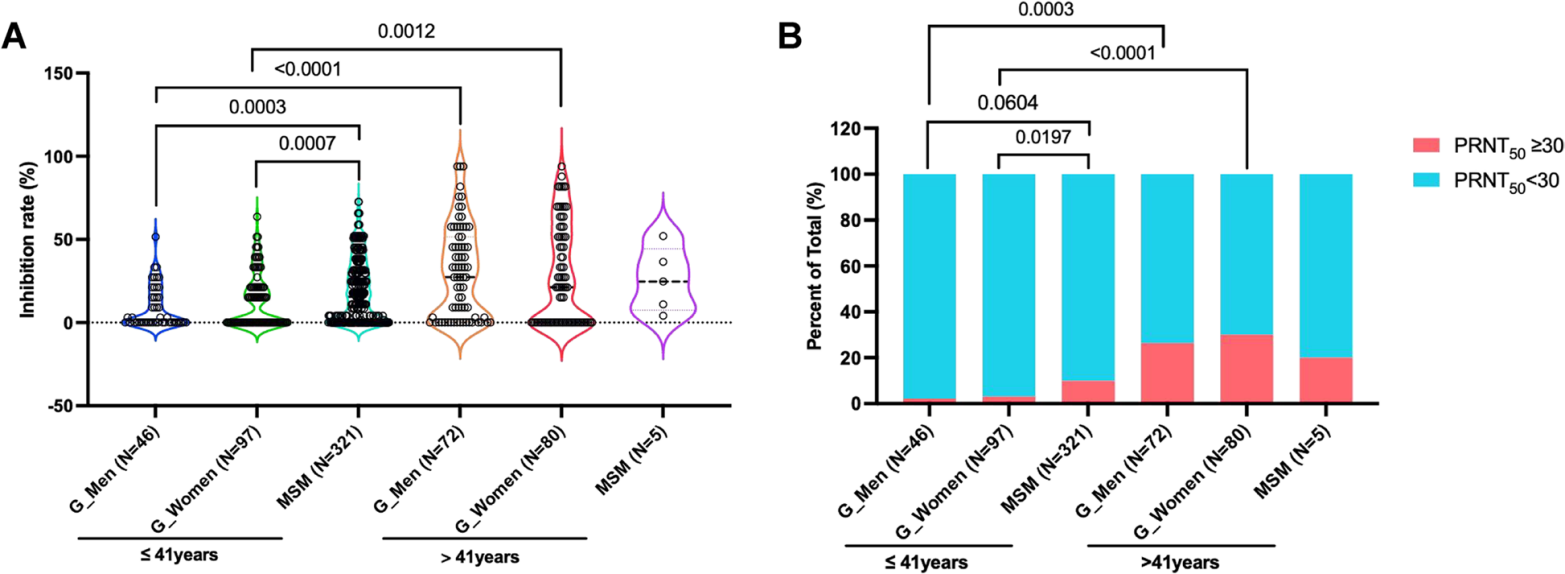
Comparisons of neutralizing antibody responses against vaccinia virus (Tiantan strain). The neutralizing antibody responses against vaccinia virus (Tiantan strain) were tested using a method of plaque reduction neutralization test. The inhibition rates (**A**) and the percentages of individuals with a PRNT_50_ ≥ 30 (**B**) were compared among groups. Statistical analyses were performed by the method of non-parametric t test (**A**) or Chi-square test (**B**)

We measured the neutralizing antibody responses against vaccinia virus (Tiantan strain) using a method of plaque reduction neutralization test. Previous studies suggested that smallpox vaccine could confer appreciable protection against MPXV [33, 34]. Therefore, we speculate that the neutralizing antibody responses against vaccinia virus may reflect the potential neutralizing activities against MPXV. Our results showed that the virus inhibition rates (%) of sera measured at a dilution of 1:30 were statistically higher among participants aged > 41 than those among participants aged ≤ 41 in the general population cohort (Fig. 2A). Meanwhile, the percentages of individuals with a PRNT_50_ ≥ 30 were also significantly higher among participants aged > 41 than those among participants aged ≤ 41 in the general population cohort (Fig. 2B). Quite unexpectedly, we found that individuals aged ≤ 41 in the MSM cohort showed significantly higher levels of neutralizing antibody responses against vaccinia virus (Tiantan strain) than individuals of the same age group in the general population cohort (Fig. 2 and Additional file 1: Fig. S4).

### The binding antibody responses against MPXV proteins were not associated with the binding and neutralizing antibody responses against vaccinia virus among participants aged ≤ 41 in both cohorts

**Fig. 3:**
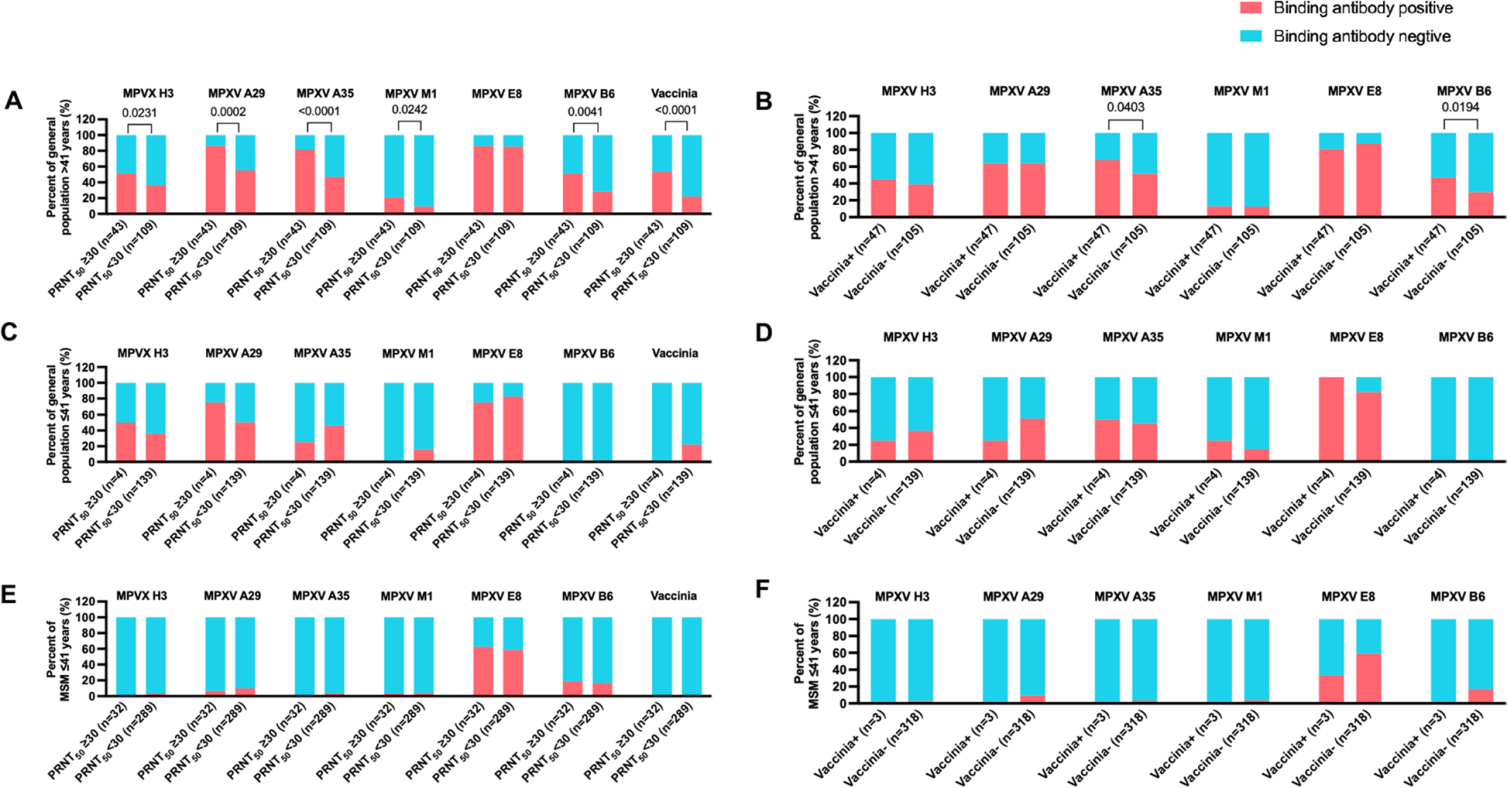
The correlation between the anti-MPXV antibody responses and the anti-vaccinia antibody responses. The positive and negative coincidence rates were analyzed by method of Chi-square test. (**A, C** and** E**) Coincidence analyses between anti-MPXV/vaccinia binding antibody responses and anti-vaccinia neutralizing antibody responses in different groups. (**B, D** and** F**) Coincidence analyses between anti-MPXV binding antibody responses and anti-vaccinia binding antibody responses in different groups. Statistical analyses were performed by the method of Chi-square test

To investigate whether the anti-MPXV antibody responses detected in the two cohorts were due to previous exposures to orthopoxviruses, we analyzed the positive and negative coincidence rates between the anti-MPXV protein antibody responses and the anti-vaccinia antibody responses. Our data showed that the positive rates of binding antibody responses against MPXV H3, A29, A35, M1, B6 and vaccinia whole-virus lysate were significantly higher among participants with a PRNT_50_ ≥ 30 in the aged > 41 group of the general cohort (Fig. 3A). In addition, the positive rates of binding antibody responses against MPXV A35 and B6 were also significantly higher among individuals with a positive anti-vaccinia binding antibody response in this group (Fig. 3B). These observations were in accord with the findings of a mouse experiment, which showed that vaccinia (Tiantan strain) induced cross-reactive antibody responses against MPXV H3, A29, A35, E8, B6 and M1 correlated significantly with the binding antibody responses against vaccinia whole-virus lysate (Additional file 1: Fig. S5). These data collectively suggested that the anti-MPXV and anti-vaccinia antibodies observed among the individuals aged > 41 might partially attribute to smallpox vaccination. In contrast, no significant association between the anti-MPXV binding antibody responses and the anti-vaccinia antibody responses was observed among individuals aged ≤ 41 in both the general population (Fig. 3C and D) and the MSM (Fig. 3E and F) cohorts, which implied that unaware exposures to orthopoxviruses might not be the main cause of the preexisting antibody responses observed among participants aged ≤ 41. Furthermore, to find out the potential cause of the higher anti-vaccinia neutralizing responses observed among the participants aged ≤ 41 in the MSM cohort, we analyzed the associations between the preexisting anti-orthopoxvirus antibody responses and the diagnosed sexually transmitted infections (STIs) in the MSM cohort. Our data showed that the positive rates of both the binding and the neutralizing antibody responses were comparable between individuals with and without diagnosed STI infections during 6 months prior to the enrollment (Table 2).

**Table 2:** Association analyses of sex transmitted infections and anti-Orthopoxvirus antibody responses among gay men aged ≤ 41 y

	**Without STI (***n***= 275)**		**With STI (*n* = 20)**		***P* value**
	**Negative (n)**	**Positive (n)**	**Negative (n)**	**Positive (n)**	
**H3 binding IgG**	267	8	19	1	0.4732
**A35 binding IgG**	266	9	19	1	0.5100
**A29 binding IgG**	250	25	17	3	0.2921
**B6 binding IgG**	267	48	17	3	0.6377
**E8 binding IgG**	110	165	8	12	0.5879
**M1 binding IgG**	263	12	20	0	0.4235
**Vaccinia binding IgG**	274	1	19	1	0.1312
**Neutralizing antibody**	246	29	18	2	0.6480

With STI, participants with diagnosed Syphilis, Gonorrhea, Chlamydia, genital herpes or genital warts during the 6 months before the enrollment. Without STI, participants without diagnosed STI during the 6 months prior to the enrollment. Participants who were not sure whether they had STIs or not during the 6 months prior to the enrollment were excluded from this analysis. PRNT_50_ ≥ 30 is classified as a positive neutralizing antibody response

Taken together, the above data indicated the anti-orthopoxvirus antibody responses observed among the participants aged ≤ 41 might not be associated with exposures to orthopoxviruses. And the higher levels of anti-vaccinia neutralizing antibody responses were not associated with STIs in the MSM cohort.

## Discussion

Orthopoxviruses have a broad host spectrum, ranging from humans to domestic and wild animals [35]. Variola, Vaccinia, VACV-like Brazilian isolates, Cowpox, Buffalopox and Monkeypox have been found to be able to infect human [36]. MPXV is highly identical to Variola at the genetic level [37–39], therefore, smallpox vaccines can provide substantial cross-protection against MPXV [33, 34, 40, 41]. It is speculated that the gradually increased monkeypox virus infection over time may be related to the increase in the proportion of the population that has not been vaccinated against smallpox [7, 42, 43]. A recent real-world study confirmed that receipt of 1 or 2 doses of a smallpox vaccine significantly reduced the risk for MPXV [44], although the duration of the protection was unknown.

Most confirmed cases in the current MPXV outbreak are men who have sex with men at a median age of 41 and it has been proposed that behavioral factors might contribute to the rapid transmission in this community [16]. While, it is unknown whether and how the preexisting immunities may influence the spread of MPXV-2022. In this study, we measured the anti-MPXV and anti-vaccinia antibody responses in a cohort of gay men and a cohort of the general population. Most of the enrolled gay men were younger than 41, which resembled the demographical characteristics of the confirmed MPXV cases. Given that China eradicated smallpox in 1979 [45] and ceased smallpox vaccination since 1981, individuals born in/after 1981 (≤ 41) should theoretically be immunologically naïve to MPXV and vaccinia virus. However, our data showed that binding antibodies against MPXV H3, A29, A35, E8, B6, M1 proteins and vaccinia whole-virus lysate could be readily detected among participants ≤ 41, although the positive rates were lower than those among participants aged > 41. These findings are in accord with previous studies demonstrating that anti-Orthopoxvirus antibodies could be detected in individuals without previous infection or vaccination [21, 23].

Moreover, our data showed that the levels of anti-vaccinia neutralizing antibody responses among the gay men aged ≤ 41 were statistically higher than those among individuals aged ≤ 41 in the general population. Unaware exposures to orthopoxviruses might not be the main cause of the anti-vaccinia neutralizing antibodies observed among participants aged ≤ 41, because the anti-vaccinia neutralizing antibody response was found to be associated with the anti-vaccinia binding antibody response in individuals who might get vaccinated against smallpox (aged > 41) but not in those aged ≤ 41. Our data also elucidated that the anti-vaccinia neutralizing antibody responses were not associated with prior STIs, which dismissed the possibility that the higher levels of neutralizing antibody responses among the gay men aged ≤ 41 were induced by STIs. A potential explanation for this phenomenon is that the pre-existing anti-MPXV antibodies might be cross-reactive antibodies induced by commensal microbial antigens or other human pathogens, which has been suggested by studies of anti-HIV [46–48], anti-SARS-CoV-2 [49, 50] and anti-influenza cross-reactive antibodies [51]. Although high levels of neutralizing antibodies against vaccinia virus could potentially provide protection against MPXV, the fact that individuals can acquire monkeypox despite smallpox vaccination [52] highlights the highly efficient transmission of MPXV through intimate contact might not be easily blocked by the pre-existing cross-reactive antibodies in real world. Further studies are needed to fully understand the relationship between these pre-existing antibodies and susceptibility to monkeypox virus infection.

Several limitations of this study should be noted. Firstly, the neutralization of monkeypox virus were not detected, because the virus was not available to us. Secondly, this study tested serum samples at a single dilution according to an experimental design of sero-prevalence investigation, hence it did not provide detail information about the titers of binding and neutralizing antibody responses. Thirdly, the purified proteins used in this study were not expressed in the same cell line which limited our confidence to compare binding antibody responses among different proteins. Nonetheless, our study showed that anti-MPXV and anti-vaccinia antibodies could be readily detected in an MSM cohort and a general population cohort. And a higher level of anti-vaccinia neutralizing antibody responses was observed among individuals who did not get vaccinated against smallpox in the MSM cohort compared to age-matched individuals in the general population cohort. Our findings highlight that the basic reproduction number (R_0_) of MPXV might be underestimated by previous studies [17, 53], because the effect of the preexisting antibody responses was not taken into consideration.

## Conclusions

Anti-MPXV and anti-vaccinia antibodies could be readily detected in an MSM cohort and a general population cohort. And a higher level of anti-vaccinia neutralizing antibody responses was observed among individuals who did not get vaccinated against smallpox (aged ≤ 41) in the MSM cohort compared to age-matched individuals in the general population cohort.

## Supplementary Information

**Additional file 1:** **Fig. S1.** The levels of background binding were low and comparable between the general population cohort and the MSM cohort. The negative controls of the ELISA assays were detected following the same procedures as being described in the Method except that the wells were not coated with antigens. The result showed that the average background binding levels of the two cohorts were comparable. Statistical analyses were performed by the method of non-parametric t test. **Fig. S2.** Comparisons of the magnitudes of binding antibodies against MPXV proteins and vaccinia whole-virus lysate. The median OD values of binding IgG responses against vaccinia whole-virus lysate and MPXV H3, A35, A29, E8, M1, B6 proteins were compared among groups. Statistical analyses were performed by the method of non-parametric t test. NC, negative control. **Fig. S3.** Comparisons of binding antibodies against vaccinia H3, A27 and A33 proteins. The positive ratesand the median OD valuesof binding IgG responses against vaccinia H3, A27 and A33 were compared among groups. Statistical analyses were performed by the method of Chi-square testor non-parametric t test. VACA, vaccinia virus. **Fig. S4.** Comparison of anti-vaccinia neutralizing antibody responses in participants aged ≤41 between the MSM and the general population cohorts. The percentages of individuals with a PRNT50 ≥30 were compared between the gay men ages ≤41 and the general individuals ages ≤41. Statistical analysis was performed by the method of Chi-square test. **Fig. S5.** Correlation analyses of the anti-MPXV binding antibody responses and the anti-vaccinia binding antibody responses in mice immunized with vaccinia virus. Female BALB/c mice were immunized intramuscularly with vaccinia virusand peripheral blood was collected at 2 weeks post vaccination. The levels of binding antibodies against MPXV H3, A29, A35, E8, B6, M1 proteins and vaccinia whole-virus lysate were compared between vaccinated and unvaccinated mice. Correlations between vaccinia induced cross-reactive antibody responses against MPXV H3, A29, A35, E8, B6, M1 and antibody responses against vaccinia whole-virus lysate were analyzed by the method of Person correlation. 

## Data Availability

The datasets used during the current study are available from the corresponding author on reasonable request.

## Abbreviations

MPXV: Monkeypox virus
MSM: Men who have sex with men
STIs: Sexually transmitted infections
