# Supplementary material for: Unexpectedly higher levels of anti-orthopoxvirus neutralizing antibodies are observed among gay men than general adult population
Source: BMC Med. 2023 May 16;21:183. doi: 10.1186/s12916-023-02872-0 (PMC10185377; doi:10.1186/s12916-023-02872-0)

Additional file 1: Fig. S1

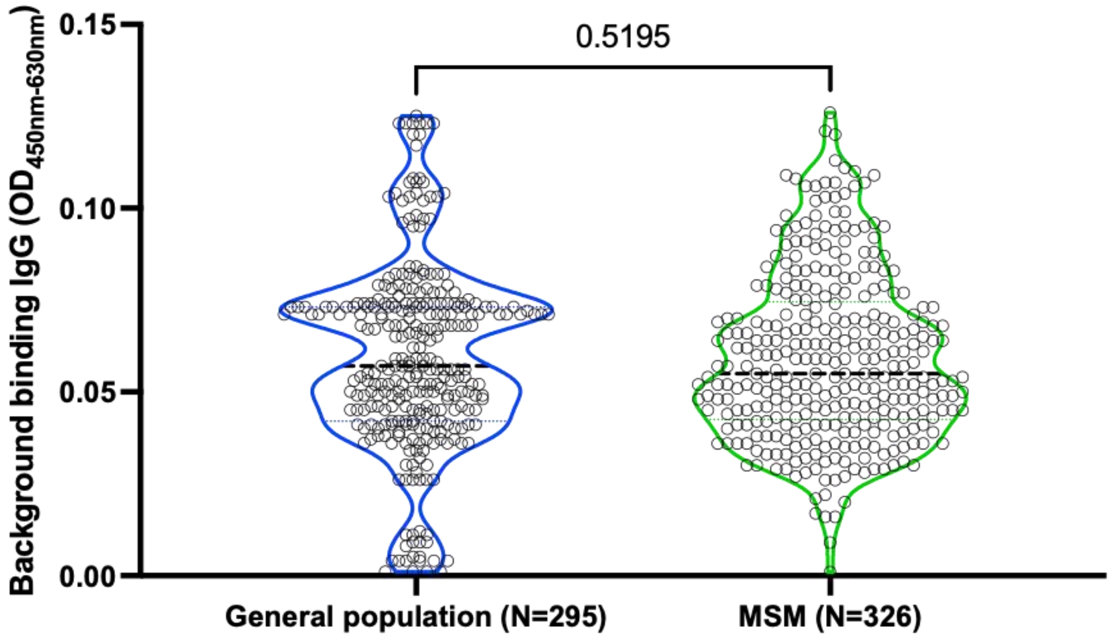

Additional file 1: Fig. S2

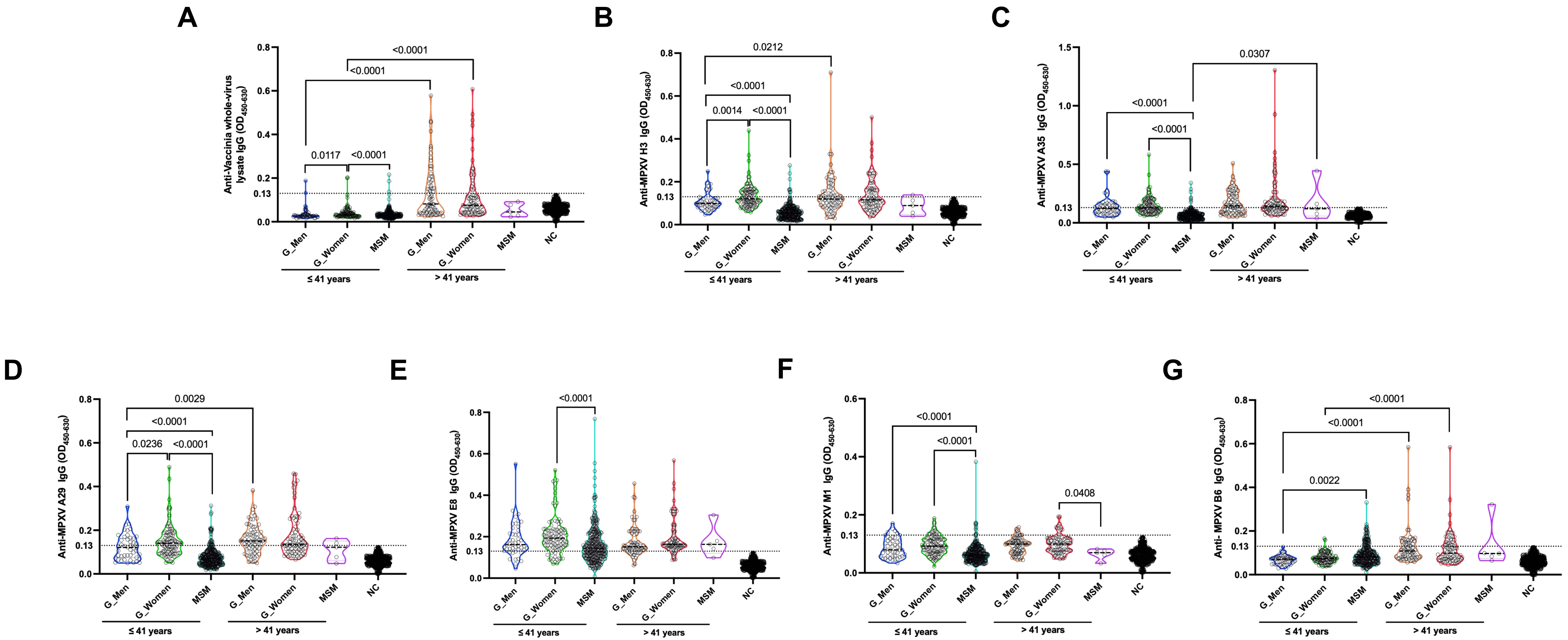

Additional file 1: Fig. S3

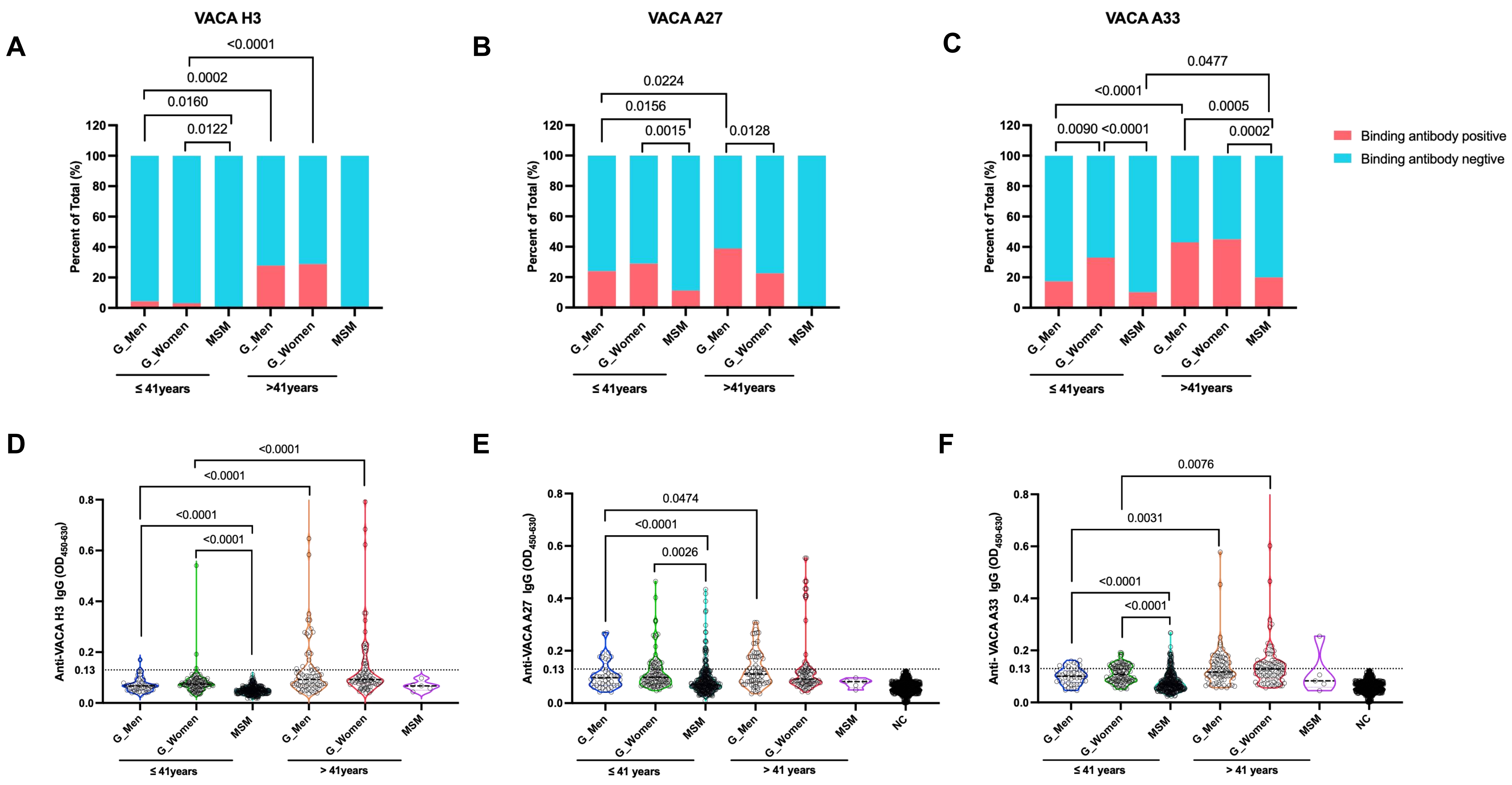

Additional file 1: Fig. S4

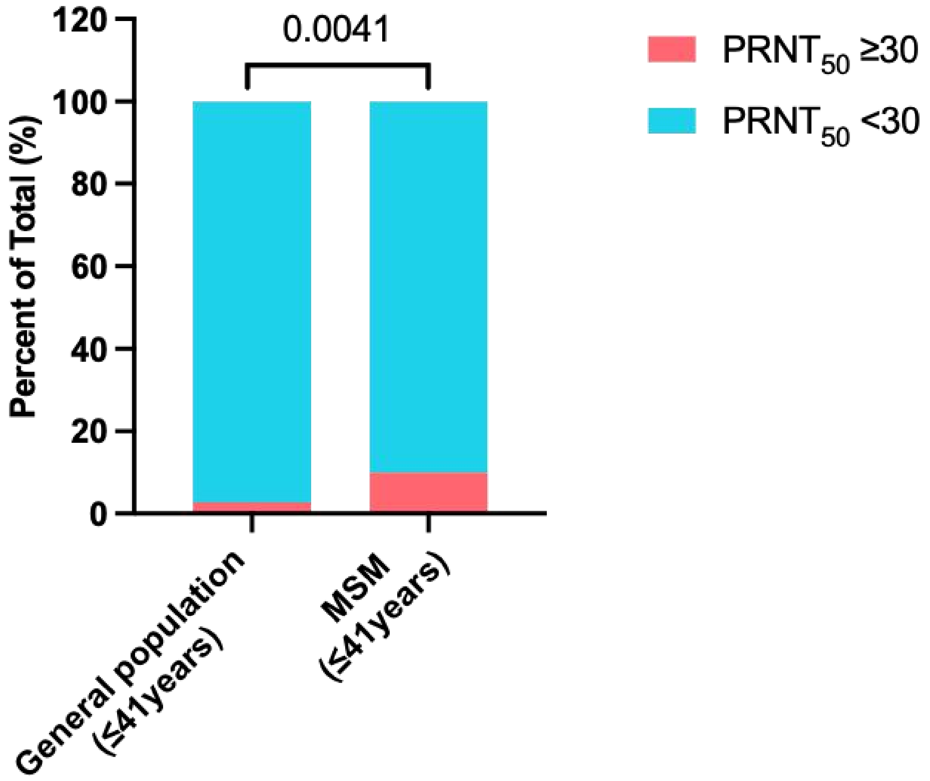

Additional file 1: Fig. S5

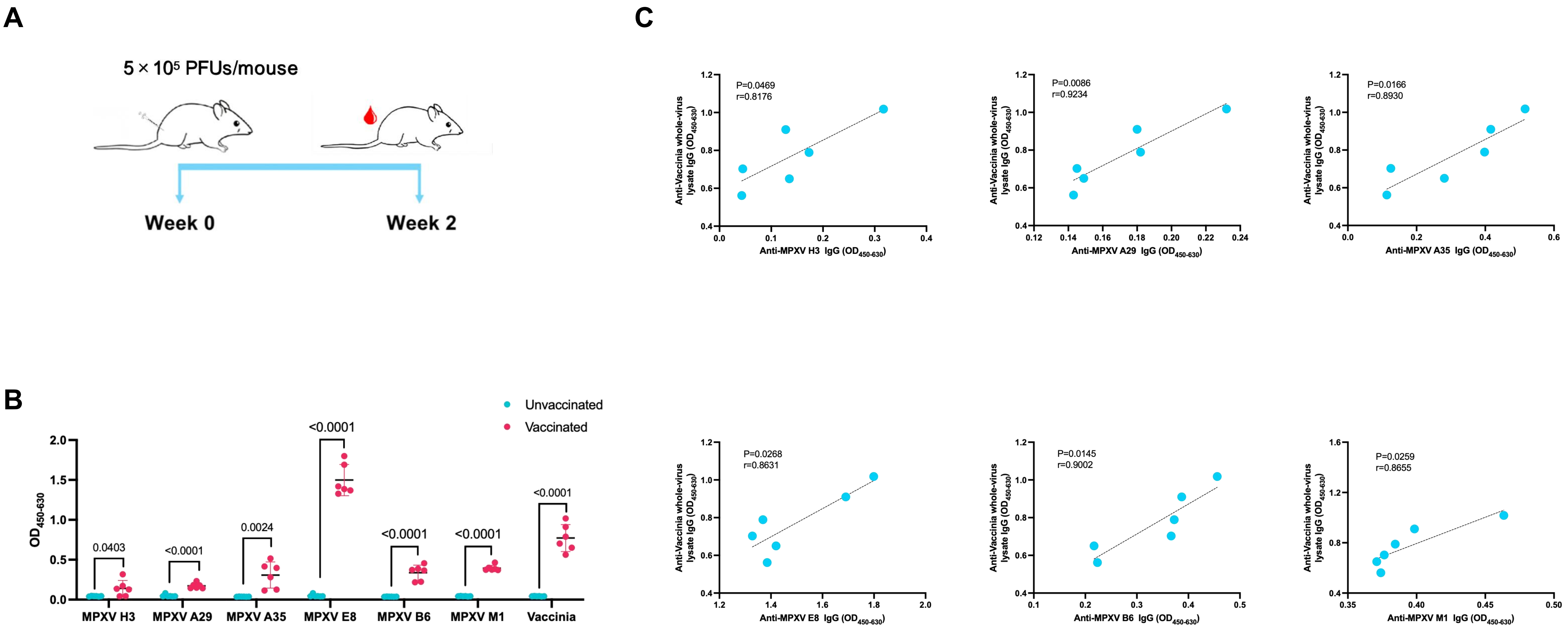

Supplement: Supplementary file 1 — Additional file 1: Fig. S1. The levels of background binding were low and comparable between the general population cohort and the MSM cohort. The negative controls of the ELISA assays were detected following the same procedures as being described in the Method except that the wells were not coated with antigens. The result showed that the average background binding levels of the two cohorts were comparable. Statistical analyses were performed by the method of non-parametric t test. Fig. S2. Comparisons of the magnitudes of binding antibodies against MPXV proteins and vaccinia whole-virus lysate. The median OD values of binding IgG responses against vaccinia whole-virus lysate and MPXV H3, A35, A29, E8, M1, B6 proteins were compared among groups. Statistical analyses were performed by the method of non-parametric t test. NC, negative control. Fig. S3. Comparisons of binding antibodies against vaccinia H3, A27 and A33 proteins. The positive ratesand the median OD valuesof binding IgG responses against vaccinia H3, A27 and A33 were compared among groups. Statistical analyses were performed by the method of Chi-square testor non-parametric t test. VACA, vaccinia virus. Fig. S4. Comparison of anti-vaccinia neutralizing antibody responses in participants aged ≤41 between the MSM and the general population cohorts. The percentages of individuals with a PRNT50 ≥30 were compared between the gay men ages ≤41 and the general individuals ages ≤41. Statistical analysis was performed by the method of Chi-square test. Fig. S5. Correlation analyses of the anti-MPXV binding antibody responses and the anti-vaccinia binding antibody responses in mice immunized with vaccinia virus. Female BALB/c mice were immunized intramuscularly with vaccinia virusand peripheral blood was collected at 2 weeks post vaccination. The levels of binding antibodies against MPXV H3, A29, A35, E8, B6, M1 proteins and vaccinia whole-virus lysate were compared between vaccinated and unvaccinated mice. Correla [file 12916_2023_2872_MOESM1_ESM.pdf]
